# Comparative Workflow Modelling of HIV Status‐Neutral Care Implementation in Six High‐Burden Jurisdictions in the United States: A Qualitative Study

**DOI:** 10.1002/jia2.70158

**Published:** 2026-10-08

**Authors:** Tongying Zhao, Laura K. Beres, Elena P. Rosenberg‐Carlson, Joseph Kenny, Wilson Gomez, Alithia Zamantakis, Cathleen E. Willging, Russell A. Brewer, Jill S. Blumenthal, Joyce L. Jones, Uyen Kao, Tequetta Valeriano, Aadia I. Rana, Sung‐Jae Lee, Maile Y. Karris, Ana M. Pachicano, Jessica Kassanits, Alison B. Hamilton, Robin G. Lanzi, LaRon E. Nelson, Lipin Lukose, Donna J. Smith, Nanette D. Benbow, Sheree R. Schwartz, Joseph G. Rosen, the NISH 001 Study Team

**Affiliations:** ^1^ Bloomberg School of Public Health Johns Hopkins University Baltimore Maryland USA; ^2^ Gillings School of Global Public Health University of North Carolina Chapel Hill North Carolina USA; ^3^ David Geffen School of Medicine University of California Los Angeles California USA; ^4^ School of Medicine Stanford University Palo Alto California USA; ^5^ School of Nursing Yale University New Haven Connecticut USA; ^6^ Feinberg School of Medicine Northwestern University Chicago Illinois USA; ^7^ Pacific Institute for Research and Evaluation Albuquerque New Mexico USA; ^8^ School of Medicine University of Chicago Chicago Illinois USA; ^9^ School of Medicine University of California San Diego California USA; ^10^ School of Medicine Johns Hopkins University Baltimore Maryland USA; ^11^ Heersink School of Medicine University of Alabama Birmingham Alabama USA; ^12^ School of Public Health University of Alabama Birmingham Alabama USA; ^13^ The Warren Alpert Medical School Brown University Providence Rhode Island USA; ^14^ School of Public Health Brown University Providence Rhode Island USA

**Keywords:** Ending the HIV Epidemic, HIV prevention, HIV treatment, implementation science, person‐centred care

## Abstract

**Introduction:**

Status‐neutral approaches to HIV treatment and prevention, which link all individuals to tailored, comprehensive services following HIV testing, are promising strategies for reducing HIV service delivery inequities and improving clinical outcomes. However, transferable, feasible models of status‐neutral service delivery are poorly understood, particularly in the United States, where implementation contexts are geographically and institutionally disparate. To guide expanded implementation, we identified difference‐making attributes of status‐neutral implementation workflows in a diverse sample of public sector‐funded HIV clinical organizations in U.S. jurisdictions prioritized in the Ending the HIV Epidemic (EHE) Initiative.

**Methods:**

In March−June 2024, we invited frontline HIV service providers across six EHE jurisdictions (Alabama, Baltimore, Chicago, Dallas, Los Angeles, San Diego) to participate in virtual focus group discussions (FGDs), segmented by ongoing or planned status‐neutral implementation. We depicted an HIV status‐neutral workflow and elicited discussants’ perspectives on (in)consistencies between the illustration and their implementation practices. Applying comparative workflow modelling, we re‐illustrated status‐neutral implementation pathways and identified workstream features distinguishing service delivery across clinical organizations.

**Results:**

We facilitated eight FGDs with 56 providers across 20 organizations. Status‐neutral implementation processes varied substantially across clinical organizations—distinguished by the roles and responsibilities of clinical (i.e. physicians, nurses) and non‐clinical (i.e. navigators, case managers) staff as well as by the sequence and timing of activities within workflows. We identified four workflow attributes that distinguished clinical organizations: (1) the timing of health insurance review (whether insurance assessments and navigation occurred earlier vs. later in the implementation pathway); (2) the magnitude of activity integration (whether services were delivered concurrently vs. sequentially); (3) the presence of implementation partnerships (whether HIV testing was conducted in‐house vs. externally); and (4) the extent of workforce task‐shifting (whether services were primarily delivered by clinical vs. non‐clinical staff).

**Conclusions:**

Workflow heterogeneities reflected jurisdictional and organizational differences in funding, staffing and infrastructure. Generalizable attributes, including the degree of workflow integration and staff role delineation, can inform scalable implementation models. Enhanced cross‐training and increased involvement of non‐clinical staff (especially in HIV prevention workflows) are critical to enable adaptable, timely and person‐centred HIV service delivery across diverse care settings.

## 1 Introduction

The United States’ HIV epidemic is characterized by drastic geographic and racialized disparities in access and outcomes [1, 2]. More than half of new HIV diagnoses occur in 1.6% of all U.S. counties, predominantly in the southeastern United States [1]. Black and non‐Hispanic White persons accounted for 43% and 26% of new HIV diagnoses, despite representing 13% and 63% of the U.S. population, respectively [1]. Further, rates of viral suppression are significantly lower among Black persons living with HIV relative to White persons [3, 4]. Pre‐exposure prophylaxis (PrEP) coverage estimates are similarly racialized, with estimated uptake in White persons (42.1%) far exceeding uptake among Black persons (5.9%) with PrEP indications [5]. These inequities in HIV prevention and treatment outcomes are underpinned by disparities in health‐related social needs (i.e. housing, employment, education), reinforced by structural racism [6, 7]. Reducing new HIV acquisitions by 90% by 2030, a stated goal of the *Ending the HIV Epidemic* (EHE) initiative, will, therefore, require adoption and sustained implementation of innovative, equity‐oriented service delivery models [8].

Efforts to reimagine HIV‐related care delivery in the United States, integrating prevention and treatment services to provide tailored and timely interventions for people at risk of or living with HIV, have gained traction in recent years [9, 10]. A status‐neutral approach to HIV treatment and prevention is one such promising strategy for addressing HIV service delivery inequities and improving clinical outcomes [9]. In the status‐neutral paradigm, a client's first touchpoint with the HIV care system is HIV testing, after which clients are connected to HIV treatment or prevention and supportive services tailored to their needs. Grounded in the principles of health equity and person‐centred care [11, 12], the status‐neutral approach seeks to avail responsive, accessible HIV‐related services that support better health for all individuals, regardless of their HIV status.

Settings where status‐neutral approaches have been piloted and up‐scaled have documented numerous advantages [13]. Within the first 3 years (2016−2019) of status‐neutral care implementation, New York City reported a 22% decrease in new HIV acquisitions [13]. Across all funded jurisdictions, rates of viral load suppression among people living with HIV on antiretroviral therapy (ART) increased 3% following status‐neutral implementation [13]. A small‐scale status‐neutral pilot in San Francisco reported not only improved HIV testing, PrEP/ART adherence and viral suppression, but also a decrease in perceived HIV‐related stigma [14]. In Louisiana, status‐neutral interventions resulted in increased skills development and employment [15].

Nevertheless, transferable, feasible models of status‐neutral service delivery are poorly understood, particularly in the United States, where implementation contexts are geographically and institutionally disparate. A recent study showed that adoption of status‐neutral HIV care among clinics funded by the Ryan White HIV/AIDS Program (RWHAP) varied across EHE jurisdictions, with clinic administrators and providers from non‐implementing facilities endorsing low perceived acceptability, appropriateness and feasibility of these service delivery models [16]. One concern is that status‐neutral approaches might direct attention and funding away from people living with HIV, potentially compromising their quality of care and treatment outcomes [17]. Another survey of 514 HIV clinics showed that clinics face tremendous resource constraints to status‐neutral implementation; 14% of clinics, for example, only had part‐time HIV providers on staff, and most lacked formal linkage mechanisms to supportive services like food and housing assistance [18]. An improved understanding of how status‐neutral‐implementing clinics in diverse practice and geographic settings have adopted and tailored the models to be contextually feasible is paramount for guiding future implementation efforts, but scholarship in this space remains limited.

Accordingly, this study aimed to identify difference‐making attributes of status‐neutral implementation workflows in a diverse sample of public sector‐funded HIV clinics in U.S. jurisdictions prioritized in the EHE initiative.

## 2 Methods

### 2.1 Study Design and Recruitment

This multisite study was led by the Network for Implementation Science in HIV (NISH), a collaborative research enterprise overseen by the HIV Implementation Science Coordination Initiative (ISCI) at Northwestern University and six partner institutions across the United States: Johns Hopkins University, the University of Alabama at Birmingham, the University of California—Los Angeles, the University of California—San Diego, the University of Chicago and Yale University [19, 20]. Beginning February 2024, we approached leadership from RWHAP‐funded HIV clinical organizations in six EHE jurisdictions (Alabama, Baltimore, Chicago, Dallas/Fort Worth, Los Angeles, San Diego) that had completed a web‐based survey of rapid HIV service delivery approaches [16] to nominate frontline HIV service providers to participate in a one‐time virtual focus group discussion (FGD). Eligible discussants included adults (aged 18+ years) employed by RWHAP‐supported clinical organizations to provide administrative, clinical, operational or other technical support to HIV prevention and/or treatment services. Only clinical organizations whose leadership self‐reported current or planned (within the next 12 months) implementation of HIV status‐neutral approaches were approached for participation.

Nominated providers were contacted via email by a trained FGD facilitator (EPR‐C, JK, JGR) or the study coordinator (AMP), who shared an information sheet detailing study objectives and procedures. Providers interested in participating were prompted to indicate their availability for a 90‐min virtual FGD; invitations with links to a secure video‐conferencing platform were then circulated to providers expressing interest and availability.

### 2.2 Data Collection

FGDs consisted of at least two clinical organizations each, with groups segmented by jurisdiction and then by current or future planned implementation of status‐neutral approaches. To stimulate rich discussion on status‐neutral implementation workflows while maintaining a non‐evaluative environment, we invited multiple organizations to participate in the same FGD—enabling discussants to compare their respective clinical implementation processes. After reading the information sheet aloud and obtaining permission to record the session, FGD facilitators—aided by semi‐structured guides (Supporting Files 1 and 2) organized around the Consolidated Framework for Implementation Research [21]—explored definitions and durations of HIV status‐neutral implementation; motivations for integrating service delivery pathways for HIV prevention and treatment; multilevel determinants (i.e. barriers and facilitators) of implementing status‐neutral models; and workflow/workforce configurations distinguishing status‐neutral approaches across settings.

To facilitate participatory dialoguing around status‐neutral workflows, we developed a harmonized flowchart illustrating potential sequences/timing and actors responsible for each component of the status‐neutral implementation process (Figure 1), which we iteratively developed by reviewing publicly available clinical implementation guidelines and eliciting input from site Principal Investigators, who are practising HIV clinicians. Starting with HIV testing, a multi‐disciplinary team facilitates health education, care planning and eligibility checking, medical and psychosocial assessment, insurance review, and linkage to care and resources. Based on the outcome of their HIV test, clients are then directed to ART or PrEP services, with providers’ ongoing support. Across FGDs, moderators projected the diagram and prompted discussants to reflect on (in)consistencies between the illustration and implementation practices at their respective clinical organizations. Discussants were also encouraged to specify adaptations to the implementation flowchart to more accurately represent the status‐neutral workflows at their respective organizations and articulate rationales for deviations from the projected flowchart.

**FIGURE 1:**
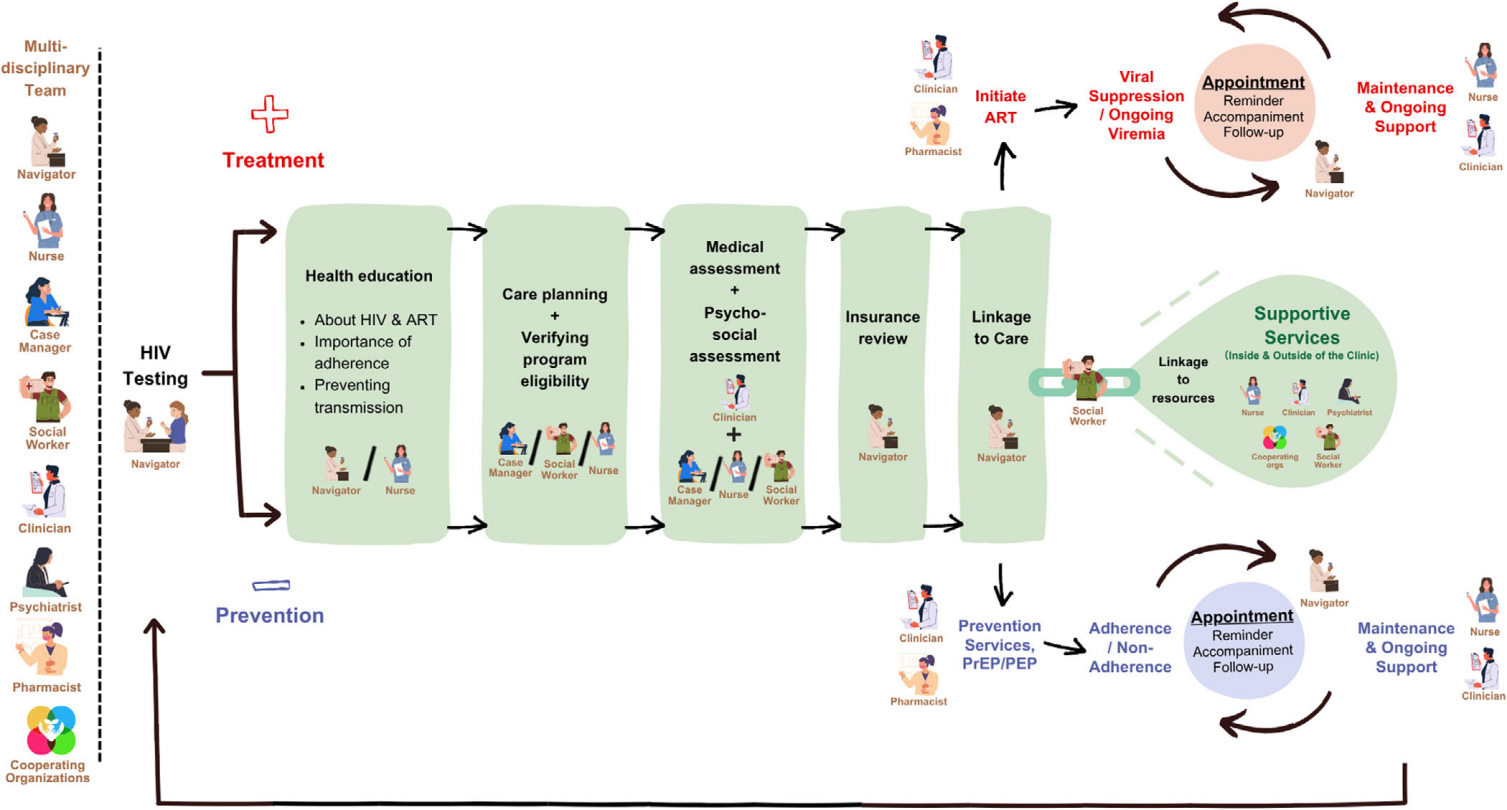
Standardized HIV status‐neutral implementation workflow. Abbreviations: ART, antiretroviral therapy; PEP, HIV post‐exposure prophylaxis; PrEP, HIV pre‐exposure prophylaxis.

Audio‐recorded FGDs were automatically transcribed within the video‐conferencing platform. Notetakers present during the FGD (TZ, WG) de‐identified and quality‐checked transcripts for accuracy, comprehension and fidelity to the recorded dialogue. Discussants received 100 U.S. Dollars, transferred via mobile payment service, for their participation.

### 2.3 Analysis

Guided by the tenets of comparative workflow modelling [22], we sought to identify attributes, or characteristics, distinguishing status‐neutral implementation processes in a heterogeneous sample of RWHAP‐funded HIV clinical organizations. We began by reading FGD transcripts line‐by‐line, inductively identifying core components of the status‐neutral workflow for each clinic. Next, the first author (TZ) leveraged discussant narratives to re‐illustrate the status‐neutral implementation pathway for each organization, comparing the sequence/timing of activities as well as implementing actors for each activity to the cross‐site workflow model displayed during FGDs (Figure 1). Clinic‐specific workflows and accompanying summary notes/memos from the first author were reviewed and discussed over multiple hour‐long virtual and multi‐day face‐to‐face convenings of the broader investigative team. These discussions facilitated pattern detection, with attention to workflow (dis)similarities, between clinical organizations. Using qualitative cluster analysis [23], we then aggregated difference‐making workflow attributes between clinical organizations into discrete elements (or clusters), which were refined through ongoing discussion among the investigative team. To enhance credibility and confirmability of these emerging workflow configurations through triangulation [24], we circulated and presented the identified difference‐making workflow attributes with study‐affiliated HIV clinicians, who further refined and affirmed the presence or absence of these workflow features as sufficiently distinguishing elements of a status‐neutral implementation pathway.

### 2.4 Ethics

Northwestern University submitted a single institutional review board (IRB) protocol through Advarra (Pro00066355), which provided a human subjects research‐exempt determination. Each site also submitted a study protocol through their governing IRBs, which exempted the study from further human subjects research oversight, reviewed and approved the study or entered into a reliance agreement with Northwestern University.

## 3 Results

Across jurisdictions, we identified and approached 54 RWHAP‐supported clinical organizations, of which 22 (41%) nominated providers to participate in an FGD. We facilitated eight FGDs (status‐neutral‐implementing: *n* = 6; non‐implementing: *n* = 2) with 56 providers across 20 clinical organizations (Table 1)—most of which were federally qualified healthcare centres (55%, *n* = 11) and implementing status‐neutral approaches (80%, *n* = 16). Participating clinical organizations were primarily constituent‐led (e.g. minority‐led and minority‐serving) but also included outreach efforts by health department‐operated clinics. The largest proportion of discussants were physicians or nurses (34%, *n* = 19), followed by service coordinators or navigators (30%, *n* = 17), case managers or social workers (29%, *n* = 16) and clinic directors/administrators (7%, *n* = 4).

**TABLE 1:** Descriptive characteristics of clinical organizations represented in focus group discussions (*N* = 20).

Characteristics (*n*, %)	Value
Jurisdiction	
Alabama	4 (20%)
Baltimore	2 (10%)
Chicago	3 (15%)
Dallas	3 (15%)
Los Angeles	4 (20%)
San Diego	4 (20%)
Status‐neutral implementation	
Current	16 (80%)
Future	4 (20%)
Organization type	
Federally qualified healthcare centre	11 (55%)
Academic medical centre	7 (35%)
Health department clinic	2 (10%)
Number of HIV service employees (median, IQR)	45 (18−75)
Number of employees licensed to prescribe ART (median, IQR)	8 (6−16)
Number of clients on ART (median, IQR)	151 (58−714)

Abbreviations: ART, antiretroviral therapy; IQR, interquartile range.

Discussant narratives revealed that status‐neutral workflows varied substantially across clinical organizations and geographic contexts, distinguished by the sequence/timing of activities within the clinical workflow as well as actors involved in their implementation. Hereunder, we summarize and present four key workflow attributes that distinguished status‐neutral implementation across clinical contexts: (1) timing of insurance review; (2) magnitude of workflow integration; (3) implementation partnerships; and (4) task‐shifting within the health workforce. We describe each of the four attributes below, accompanied by provider narratives and illustrations—highlighting where workflows diverged most prominently, as well as principal drivers of these implementation heterogeneities.

### 3.1 Timing of Insurance Review

Discussant narratives revealed heterogeneities in the timing at which providers conducted insurance assessments, whether at the beginning of the clients’ visits or upon linkage to care (Figure 2). Decisions surrounding the timing of insurance assessments reflected considerations of insurance or supporting programme eligibility, practicality and early problem detection.

**FIGURE 2:**
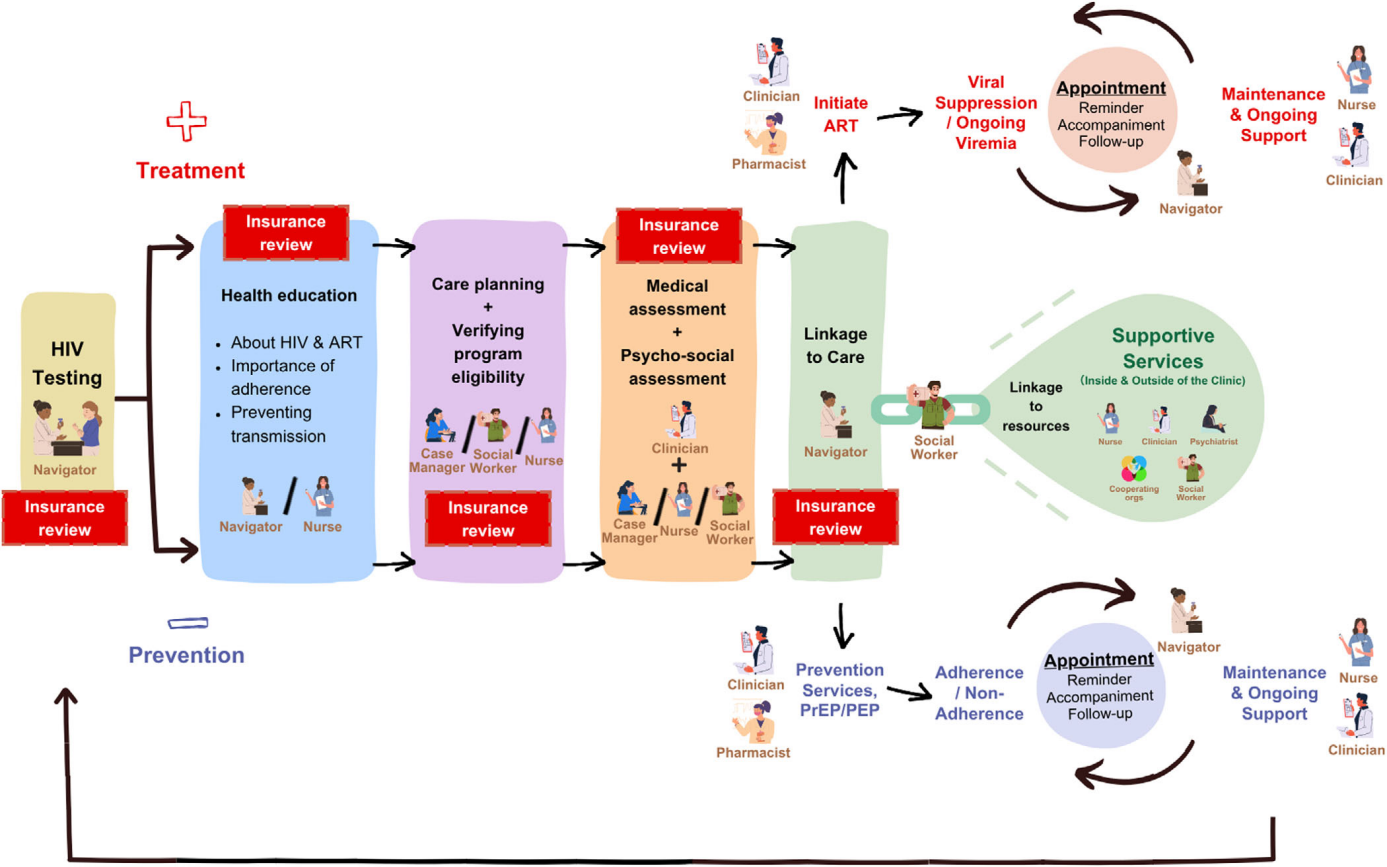
Difference‐making attribute: Timing of insurance review. Abbreviations: ART, antiretroviral therapy; PEP, HIV post‐exposure prophylaxis; PrEP, HIV pre‐exposure prophylaxis. Rather than being a stand‐alone step before linkage to care, insurance review can occur at multiple touchpoints along the prevention and care continua, from testing to linkage to care.

Many providers confirmed, in alignment with Figure 1, that insurance review was among the first steps in the status‐neutral workflow. Some referred to funding sources and billing considerations of the clinic as the principal driver of assessing clients’ insurance status earlier in the workflow, especially in the context of PrEP service delivery (which sometimes required supplemental efforts for resource mobilization).

*We try to problem solve before we get to the medical assessment. If someone's HIV‐positive, [RWHAP] is something we can get on the same day for them…But for someone on PrEP, during that care‐planning stage, we work out all the insurance stuff before they even meet with the clinician. In that way, we've worked out some of the barriers before they receive a prescription*. (Case Manager, San Diego)

Others noted that situating insurance review earlier in the status‐neutral workflow reflected attempts to troubleshoot potential bottlenecks to long‐term care continuity, especially those related to economic stability and other social determinants of health. Because financial assistance is oftentimes a prerequisite to PrEP or ART continuity, especially for uninsured or under‐insured clients, earlier insurance review helped ensure that costs related to PrEP or ART care were covered upfront.

*The linkage specialist usually does insurance screening for newly diagnosed patients before they come in for an appointment because paying for the services always stresses the patients out, which could be a barrier for them to engage in care*. (Service Coordinator, Dallas/Fort Worth)

Discussants further clarified that earlier insurance reviews could be used to screen clients for service eligibility and proactively avoid administrative/clinical procedures that would inevitably have to be completed at another clinic.

*We want to know their insurance before we do an assessment. If we already know they're not going to be able to go to [our clinic] because of insurance, they must go somewhere else. It can be traumatizing for a patient to go through a whole assessment at one place and then do it all over again at another place*. (Clinic Administrator, Baltimore)

At some clinical organizations, discussants alluded to specific circumstances that necessitated insurance review to occur at multiple touchpoints along the HIV prevention and care continua. In many cases, repeated insurance reviews implicated multiple clinical and non‐clinical staff at different inflection points in the status‐neutral workflow, from appointment scheduling to prescribing.

*A lot of our patients don't have insurance, so one is accessing the different safety nets that are available for patients to get their medication, whether ART or PrEP…It's a back and forth with the pharmacy to see whether their [insurance] is going to pay for this medication…It takes not only the navigator, but also the pharmacist to help us with that*. (Nurse, Dallas/Fort Worth)

In other instances, especially for clinics specializing in populations requiring additional privacy protections (e.g. adolescents), repeating insurance assessments throughout the status‐neutral workflow enabled providers to develop more responsive care plans for their clients:

*A lot of them [adolescents] are under their parents’ insurance. When we're talking about things like PrEP, we're really concerned about confidentiality, so we'd like to know upfront how we can come up with a care plan with what is accessible to them and things that they'll feel safe with. That's why we ask [about insurance] upfront…And then towards the end, as we're about to do linkage [to care], that's usually where we start navigating…them to insurance*. (Nurse, Baltimore)

### 3.2 Magnitude of Workflow Integration

Most clinical organizations, especially larger and well‐resourced status‐neutral‐implementing clinics, were characterized by “vertical staffing” approaches to service delivery, whereby key activities within the status‐neutral workflow were conducted sequentially. Such approaches involved highly protocolized handoffs of clients from one department or staff member to another. As discussants described, vertical staffing can support a structured sequence of client appointments. Each step in the prevention and care continua, from testing to prescribing, can be assigned to specialized personnel, promoting consistent and efficient provision of quality services. Different personnel form a cohesive system of support, which was largely welcomed by clients. Rather than functioning as isolated encounters, services provided by different departments were complementary to each other, with each handoff building on the previous ones. Vertical staffing also allows clients more time to process information, build rapport/trust with the care team and prepare themselves for treatment initiation.

*For the most part, most patients do welcome [vertical staffing] because you are meeting people from every [department]: the nutritionist, mental health, substance abuse, and the case manager. Because everyone is on the same page, I think that helps support the patient very well and keeps them on track*. (Clinic Administrator, Alabama)

However, discussants pointed out that the various steps and handoffs could increase provider workloads and heighten the risk of client attrition. This staffing approach was also sometimes associated with more complex referral/handoff procedures and workflow inefficiencies, particularly when communication between different departments or staff is ruptured.

*There are several people involved [in the status‐neutral workflow], so it gets confusing about who needs to notify whom, and what role I am supposed to play. Usually, for example, if it's an in‐house diagnosis, if they have a positive rapid test, I do all the intakes. I'm supposed to be notified that this person is coming back in a week to receive a confirmatory test result, and then I plan to meet with them that same day…But sometimes I get left out…I think we need to have a better communication flow throughout the process*. (Social Worker, Alabama)

By comparison, other clinical organizations reimagined the status‐neutral workflow as an integrated, or parallel, process (Figure 3)—primarily to ensure that clients complete all relevant activities from testing to service linkage, within a single clinical encounter lasting, at most, a few hours. One discussant described that within a single clinical encounter, a case manager and a nurse responsible for linkage to care would work collaboratively with the client to review their insurance, make alternative plans if they were uninsured, conduct requisite medical and psychosocial assessments, and schedule an appointment with a clinician for PrEP or ART dispensation.

**FIGURE 3:**
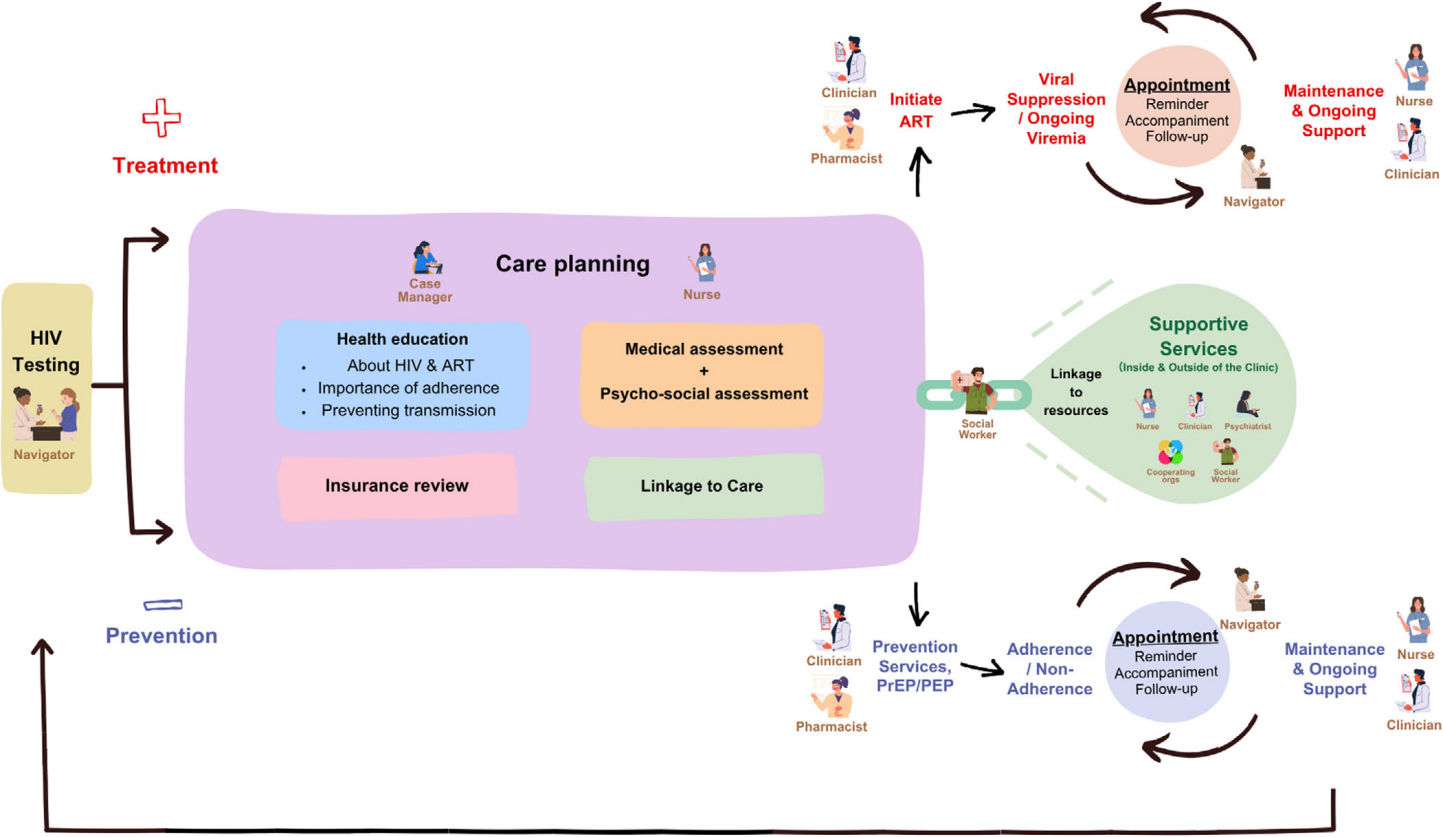
Difference‐making attribute: Magnitude of workflow integration. Abbreviations: ART, antiretroviral therapy; PEP, HIV post‐exposure prophylaxis; PrEP, HIV pre‐exposure prophylaxis. Rather than having multiple handoffs between personnel from health education to care planning, assessments and linkage to care, these steps are integrated into a single care planning appointment managed by a case manager and a nurse.

*We try to make the time to complete this “arc” as short as humanly possible…You don't want to hold onto people for 8 hours*. (Nurse, Baltimore)

Clinical organizations with more integrated service delivery typically assigned personnel to multiple activities/tasks within the status‐neutral workflow to optimize efficiencies. Participants pointed out that such “horizontal staffing” could streamline the activities to reduce delays in care provision and attrition along the HIV treatment or prevention continua. At the same time, clients may be less overwhelmed by all the different services and activities if their care was overseen by a smaller number of providers.

*A lot of these steps [in the status‐neutral workflow] are handled by one person. I could be your insurance navigator. I could be your health educator. I'm your case manager. I'm your linkage to resources. If someone comes in, [a service coordinator] can see them and help with all the steps, so it's not necessarily separate people. Not knowing who to send people to delays care and getting patients connected to resources*. (Case Manager, Chicago)

For some clinical organizations, horizontal staffing was the product of resource constraints, including insufficient staff to absorb discrete activities or roles within the workflow. This pattern was most prominent among non‐implementing clinics. To reduce workloads and maintain focus, some clinical organizations still sought to expand their staffing to include individuals who could fulfil different, albeit complementary, activities in the status‐neutral workflow.

*We have fewer staff. We have fewer clients. This [standardized status‐neutral workflow] looks very similar to how we do this here…Some people do extra roles, as we don't have all these employees, but the process is very similar*. (Social Worker, Alabama)

While staffing integrations were perceived to improve efficiencies in service delivery, discussants noted that the success of this staffing model hinged upon the competencies of one or a few providers who could master all aspects of the status‐neutral workflow. Challenges associated with this included overburdened staff and potential losses of institutional knowledge and resources attributed to staff turnover.

*It all falls on one person. You have certain people in the clinic who are repositories of information, and then that gets locked up in that person. If that person leaves, you'll lose institutional knowledge. And because this one person can do all that, they pull them in 3,000 different directions. They're diluting all the energy*. (Navigator, Chicago)

### 3.3 Implementation Partnerships

Most clinical organizations described in‐house HIV testing efforts (i.e. implemented by staff from the same organization). A few reported that because of funding constraints, HIV testing was offered through partner emergency departments or sexually transmitted infection (STI) clinics, before the clients were linked to providers implementing HIV‐specific services.

*We have routine opt‐out testing. For anyone who comes in for services, HIV testing is offered unless they already have an HIV diagnosis…It is just standard for everyone. It's kind of like our screening tool*. (Case Manager, San Diego)

Rather than having in‐house HIV testing as the only entry point into the prevention and treatment pathways, clients may enter via community outreach events and testing in emergency departments (Figure 4). Some clinical organizations reported primarily implementing HIV testing through community‐based outreach, while others described formal partnerships with other community‐based organizations (CBOs) implementing HIV testing. Specifically, non‐implementing clinics rarely conducted HIV testing themselves due to funding constraints and instead primarily received client referrals. Both approaches expanded points of entry into the status‐neutral workflow. One discussant explained that having HIV testing widely available and visible in different settings would help reduce HIV‐related stigma, promoting equity in access to HIV and complementary services, especially for multiply marginalized populations.

**FIGURE 4:**
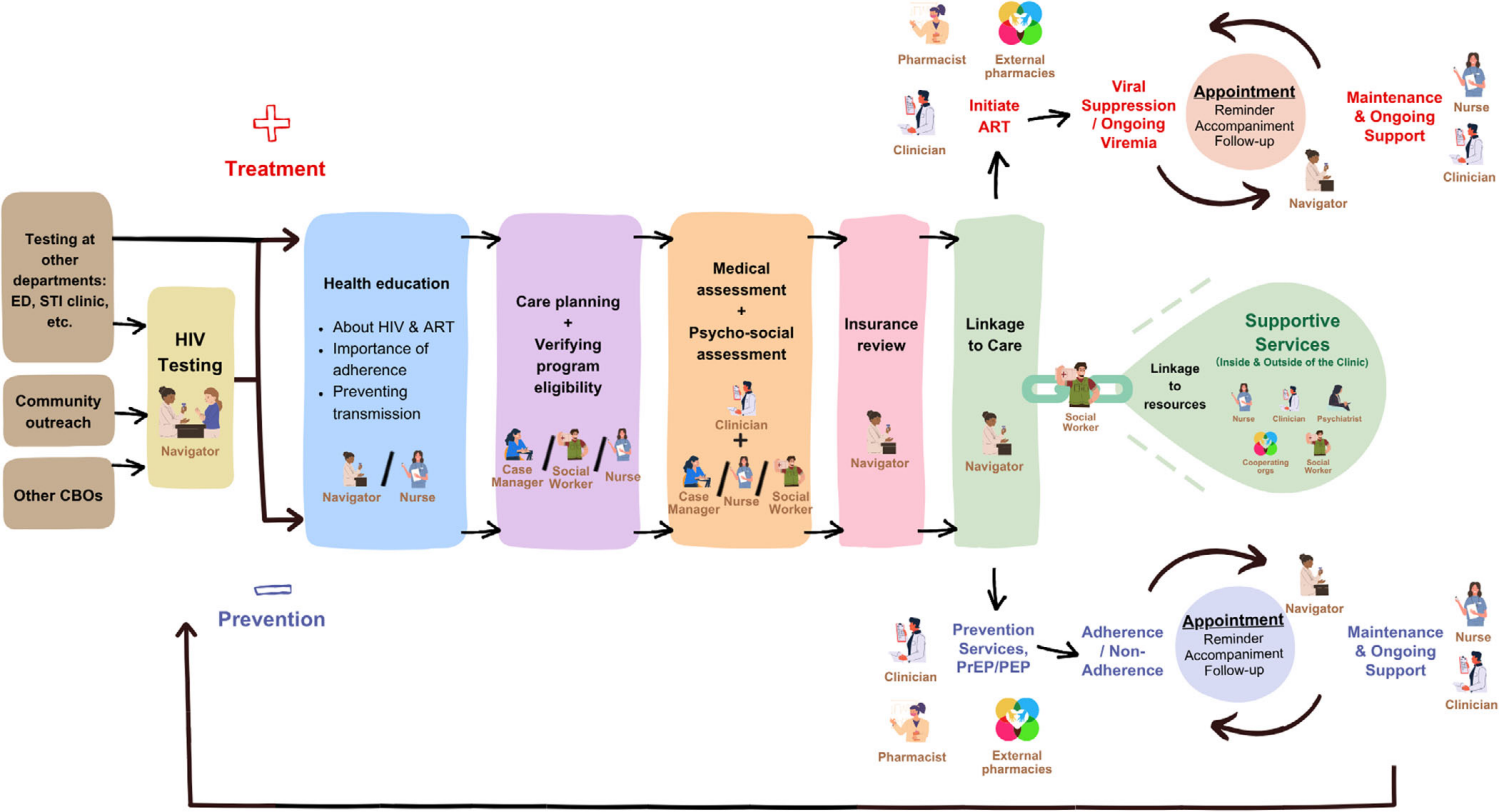
Difference‐making attribute: Implementation partnership. Abbreviations: ART, antiretroviral therapy; PEP, HIV post‐exposure prophylaxis; PrEP, HIV pre‐exposure prophylaxis. Rather than having in‐house HIV testing as the only entry point into the prevention and treatment pathways, clients may enter via community outreach events and testing in emergency departments. Additionally, PrEP/PEP and ART might be dispensed at both in‐house and external pharmacies.

*If we start making it that you can get testing anywhere, it reduces the stigma. We can stop stressing the prevention versus HIV [treatment]. We're all just people trying to get services like housing…We all have the same needs*. (Nurse, Baltimore)

In addition to testing services, discussants described differences in settings where PrEP and ART were dispensed within the status‐neutral workflow. While some clinical organizations had in‐house pharmacies that offered both ART and PrEP, others mentioned cooperating with external pharmacies to facilitate medication access (Figure 4). A few providers noted that clients’ insurance might restrict them to certain pharmacies, with those initiating PrEP facing potentially greater barriers.

*We are usually able to do [same‐day PrEP]. But occasionally, if someone does have insurance, but their insurance restricts them to certain pharmacies, they may not get out with the medications [the same day]*. (Physician, San Diego)

### 3.4 Task‐Shifting Within the Health Workforce

Status‐neutral implementation across clinical organizations was further distinguished by the magnitude of oversight by clinicians, with some clinical organizations describing status‐neutral workflows where clinicians were present at each stage (Figure 5), while others reported more involvement or leadership by non‐clinical actors. Clinician‐dominated workflows were observed in some relatively small organizations with fewer staff, requiring clinic leadership to participate in all components of the status‐neutral workflow. In many circumstances, clinical organizations required that specific steps like confirmatory HIV testing and PrEP/ART prescription must be implemented by a licensed clinician.

**FIGURE 5:**
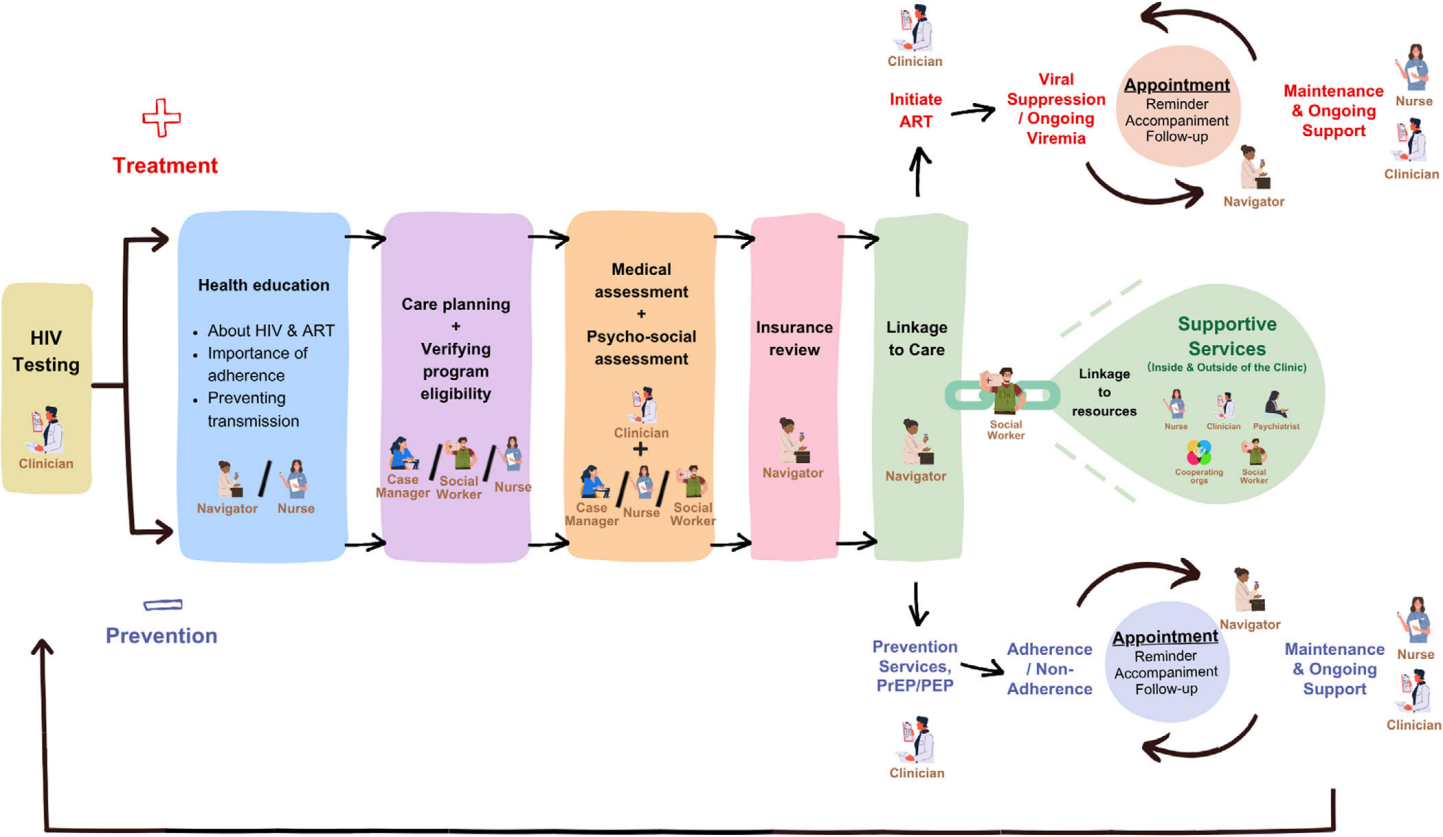
Difference‐making attribute: Task‐shifting within health workforce. Abbreviations: ART, antiretroviral therapy; PEP, HIV post‐exposure prophylaxis; PrEP, HIV pre‐exposure prophylaxis. Rather than involving non‐clinical actors such as social workers in the care pathways, the workflow shown here is dominated by clinicians and nurses.

*If there's a person who tests positive, something that is relatively new to us that I find unfortunate is that there was a time when…we could have our nurse do the blood draw for the confirmatory test. But we can't do that now…So, if the provider is not here, because we have only one provider, we have to call the Health Department to take over*. (Clinic Administrator, Alabama)

Other clinical organizations, however, involved social workers and other non‐clinical actors in these steps, including allowing them to dispense ART/PrEP. This was perceived to increase service provision agility and accessibility, as clients can proceed to subsequent stages of the treatment or prevention continua without requiring oversight or involvement of a licensed provider.

*What isn't difficult is getting [ART] prescribed to the pharmacy [or] getting the correct orders written for labs. Those are all things that could be done without seeing the provider…In our system, it's a [licensed vocational nurse] who carries out these steps, but it really is who you feel comfortable training to that level*. (Service Coordinator, Los Angeles)

## 4 Discussion

We identified four key workflow attributes—timing of insurance review, degree of workflow integration, implementation partnerships and task‐shifting within the workforce—that distinguished the implementation of HIV status‐neutral approaches across diverse public sector‐funded HIV clinical organizations. Such variations reflect how clinical organizations have adapted status‐neutral approaches to their specific context of funding structure, resource availability and relationships with external entities like CBOs—highlighting the potential and feasibility of contextually tailored status‐neutral implementation.

Rather than being a stand‐alone step before linkage to care, insurance review can occur at multiple touchpoints along the prevention and care continua. Early insurance review in the status‐neutral pathway was acknowledged as an important way to troubleshoot socio‐structural barriers to ART/PrEP initiation and circumvent duplicative administrative and clinical procedures. This is consistent with studies emphasizing insurance coverage as a political/legal determinant of HIV outcomes in the United States [25, 26], with under‐ or uninsured persons being less likely to be retained in HIV care, report optimal ART adherence and achieve viral load suppression [26, 27]. Uninsured or under‐insured people seeking PrEP or post‐exposure prophylaxis (PEP) might face additional challenges, as many RWHAP‐funded programmes provide a safety net for people living with HIV but offer modest‐to‐no support for prevention services [25]. Given recent U.S. health‐related funding cuts, growing financial pressure and funding uncertainties could limit organizations’ capacity to provide insurance navigation services [28]. Accordingly, clinical organizations with and without resources to cover expenses for uninsured and under‐insured clients may be incentivized to administer insurance assessments earlier in the status‐neutral pathway, either to identify and match clients in need of financial support with eligible resources or direct them to other capable clinics. Moreover, insurance assessments might be repeated several times throughout the workflow, as multiple clinical and non‐clinical staff collaborate in developing responsive care plans for clients, especially those requiring additional privacy protections who may delay or decline HIV services to avoid inadvertent disclosure of sexual identities or behaviour to caregivers [29, 30].

Clinical organizations also exhibited variable magnitudes of workflow integration, ranging from sequential to convergent service delivery. Many clinical organizations provided different services in a sequential manner, with clients receiving discrete services from different departments or personnel. Such an approach may permit clients more time to prepare for PrEP/ART initiation and receive services from a highly specialized, albeit siloed, interdisciplinary team of providers. Multidisciplinary clinical teams may better address medical and social concerns for people living with HIV, providing clients with a broader, more tailored support network [31, 32]. However, our study found a major challenge with vertical staffing models to be client attrition and workflow inefficiencies due to multiple inter‐departmental handoffs. Other resource‐constrained clinical organizations were more likely to have integrated status‐neutral workflows—whereby health education, care planning, clinical assessments and service linkage are all integrated into a single clinical encounter with 1–2 staff members. Despite their limitations (e.g. overburdened staff, loss of institutional knowledge and expertise with staff turnovers), such “one‐stop shop” models might significantly reduce client wait times and increasing care satisfaction among people seeking HIV prevention services, as evidenced in Kenya [33]. Overall, clinical organizations embraced staffing models that best fit their resource capacity and supported clients’ needs. Enhancing inter‐departmental communication in more siloed workflows is imperative to minimizing bottlenecks during client handoffs. However, in the context of U.S. funding cuts since 2025, an increasing number of organizations could face resource shortfalls—driving clinics towards more integrated workflows. Thus, cross‐training, shared documentation and succession planning might help with the potential transition, reducing dependency on specific individuals in integrated status‐neutral workflows.

Partnerships with external entities also emerged as a difference‐making attribute of status‐neutral implementation pathways, reflected by different arrangements of testing and medication dispensing. While most clinical organizations had access to in‐house HIV testing resources, including through affiliated emergency departments and STI clinics, others relied on community outreach teams or formalized partnerships with CBOs. Both strategies have attracted increasing attention. For instance, STI clinics appeared to be successful at serving people disproportionately impacted by HIV and identifying new HIV diagnoses in the United States [34, 35], expanding testing services to meet EHE goals [36]. Meanwhile, reaching people outside of healthcare settings through targeted mobile and outreach testing has been a key strategy of U.S. HIV prevention service provision for decades [36], expanding entry points into the status‐neutral workflow. While participants did not elaborate on the specific types of CBOs, greater attention to these organizations in future work may help identify collaboration opportunities. Beyond HIV testing services, medication dispensing was another area of cooperation reported by some clinical organizations. In high‐burden jurisdictions in the southeastern United States, cooperative agreements with pharmacies to deliver PrEP expanded accessibility in the most underserved geographies [37]. Pharmacy‐led PrEP services are also highly acceptable to clients and implementation constituents in the United States [38]. Clear referral protocols and data‐sharing systems, as well as enhanced support from policymakers and funders, could strengthen partnerships between HIV clinics and other organizations providing testing, ART/PrEP dispensation and supportive services. In turn, this could then expand entry points into HIV care systems, increase accessibility to medication dispensing venues, and enhance HIV treatment and/or prevention service continuity.

Status‐neutral workflows were further distinguished by task‐shifting within the healthcare workforce. Clinician‐dominated workflows were most frequently observed in smaller clinical organizations, where resource constraints prevented them from building larger teams with full‐ or part‐time staff in supporting roles, while better‐resourced entities engaged social workers and other non‐clinical actors in medication dispensing (resulting in more flexible service provision models). Intensified needs for task‐shifting emerged during COVID‐19 in diverse areas of health services, including HIV [39]. With adequate training and cooperation, task‐shifting may strengthen workforce resilience and capacity, improving access to HIV services, and streamlining care delivery [39]. Our findings similarly suggest that task‐shifting could improve efficiencies and capacities in the HIV service provision workforce, given that clinics provide sufficient training and supervision mechanisms to ensure that non‐clinical staff are well‐equipped to absorb clinical responsibilities while adhering to care quality standards.

Our study has limitations. Although we captured perspectives from diverse frontline HIV service providers, perspectives from CBO representatives and clients initiating ART or PrEP within the status‐neutral paradigm are absent. CBO and client perspectives/experiences have rarely been explored in existing research, with only one study showing that marginalized clients highly valued access to resources including food and housing, alongside health screening and medication refills [40]. Future studies should address these limitations by examining client outcomes and interrogating client perspectives on status‐neutral implementation. Mixed‐methods research linking these distinct workflow models to outcomes like linkage, retention, viral suppression, stigma reduction and health equities would further strengthen the evidence base. Further, our unit of the analysis was the clinical organization, rather than the focus group; we did not undertake a focus group interaction analysis. Our study was also restricted to RWHAP‐funded clinical organizations, which generally have access to more financial resources for service provision relative to non‐federally funded clinics [41, 42], in six jurisdictions; accordingly, findings may not reflect the full constellation of difference‐making attributes across status‐neutral models in the United States. Moreover, while the largest fraction of RWHAP grantees are federally qualified healthcare centres (consistent with the composition of clinics included in the present study), clinics in the present study may not be representative of the HIV service delivery landscape in these EHE jurisdictions. Finally, while we sought to cultivate a non‐judgemental and open discussion environment, discussants may have been reticent to transparently share site‐specific implementation challenges among other service providers, as we could not guarantee confidentiality in the focus group setting.

## 5 Conclusions

This study demonstrates that differences in how clinical organizations implement status‐neutral HIV care reflect practical adaptations to both system constraints (i.e. resource and staffing limitations) and the needs of their client populations. Clinical organizations varied in how they sequenced key activities like insurance review, assigned staff roles/responsibilities, integrated different services and partnered with external organizations. To scale status‐neutral care equitably, whether in the United States or other middle‐ and high‐income countries, funders and policymakers should prioritize dedicated financial support for prevention services, incentivize early and repeated insurance review, and invest in cross‐training of non‐clinical staff. In the United States and abroad (including low‐ and middle‐income countries), strengthening partnerships with pharmacies and CBOs can expand clinical entry points, while intentional task‐shifting can bolster workforce agility and competencies.

## Author Contributions

LKB, EPR‐C, AZ, CEW, ABH, RGL, NDB, SRS and JGR conceptualized the present study and developed its guiding methodology. LKB, RAB, CEW, ABH, RGL, LEN, NDB and SRS acquired funding to support the present study. RAB, JSB, JLJ, UK, TV, AIR, SL, MYK, LEN, NDB and JGR recruited participants for data collection. EPR‐C, JK and JGR collected data, with logistical and data management support from TZ, WG, AMP and JK. TZ led data analysis, with support from EPR‐C, WG, AZ, AMP, LL and DJS; input from CEW, JSB, JLJ, UK, TV, AIR, S‐JL, MYK, RGL, LEN and NDB; and oversight from LKB, SRS and JGR. TZ prepared the first draft of the manuscript. All authors participated in data interpretation, contributed to and reviewed the manuscript, and had final responsibility for the decision to submit the manuscript for publication.

## Funding

This study was funded by the following National Institutes of Health‐funded Centers for AIDS Research and AIDS Research Centers: the Center for HIV Identification, Prevention, and Treatment Services at the University of California, Los Angeles (P30MH058107); the Center for Interdisciplinary Research on AIDS at Yale University (P30MH062294); the Johns Hopkins University Center for AIDS Research (P30AI04189); the San Diego Center for AIDS Research (P30AI036214); the Third Coast Center for AIDS Research (P30AI117943); and the University of Alabama at Birmingham Center for AIDS Research (P30AI027767). The authors also acknowledge support from the National Institute of Allergy and Infectious Diseases (R24AI191821, R24AI191842, R24AI191865, R24AI191868, R24AI194681, R24AI194905). LKB and JGR report additional funding from the National Institute of Mental Health (K01MH130244, R25MH083620). SRS acknowledges support from the Center for HIV and Mental Health Stigma Elimination Strategies (CHIMES), a programme of the National Institute of Mental Health (P30MH136919).

## Conflicts of Interest

The authors have no conflicts of interest to disclose.

## Supporting information




**Supporting File 1**: Clinics Implementing Status‐Neutral Approaches

**Supporting File 2**: Draft 14.0: Clinics NOT Implementing Status‐Neutral Approaches

## Data Availability

The de‐identified focus group transcripts are not publicly available but can be made accessible, upon reasonable request, by contacting the corresponding author, Dr. Joseph G. Rosen (joseph_g_rosen@brown.edu).
