# Supplementary material for: Comparative Workflow Modelling of HIV Status‐Neutral Care Implementation in Six High‐Burden Jurisdictions in the United States: A Qualitative Study
Source: J Int AIDS Soc. 2026 Oct 8;29(Suppl 5):e70158. doi: 10.1002/jia2.70158 (PMC13650420; doi:10.1002/jia2.70158)
Supplement: Supplementary file 1 [file JIA2-29-e70158-s001.docx]

NISH 001 Focus Group Discussion Guide

**Draft 14.0: Clinics NOT Implementing Status-Neutral Approaches**

**When ready to begin: ADMIT DISCUSSANTS FROM THE WAITING ROOM**

Hi, my name is ______ and I’m [**STATE AFFILIATION WITH THE STUDY]**. I’ll be facilitating our discussion today. I will turn it over to our notetaker _______ to introduce themselves. [**NOTETAKER INTRODUCES THEMSELVES**].

Thank you all for agreeing to take part in this focus group. We appreciate your willingness to participate and the time you are taking out of your day to do so. You have all been invited to participate in today’s focus group because you are providers at clinics in [**LOCATION**] that are interested but not yet implementing HIV status-neutral approaches.

This focus group is part of a larger pilot study to understand interest and readiness within Ryan White-funded clinics to implement rapid ART, same-day PrEP, and status-neutral approaches. Before we begin, I want to remind everyone that by joining today’s focus group, you have agreed to the procedures outlined in the study information sheet and you are comfortable with today’s session being recorded. This recording will be deleted once we obtain a transcript of the conversation and de-identify all personal information. We also ask everyone to respect each other’s privacy and to treat today’s conversation confidentially.

To quickly set some ground rules, we encourage everyone to stay on camera and unmuted, even when you are not speaking, if you are in a quiet place and comfortable doing so. We also encourage everyone to participate today through spoken dialogue, rather than using the Zoom chat. We will also refer to you by the name indicated on your Zoom profile; please update your Zoom icon to reflect the name you would like to be called during today’s discussion. Our notetaker ______ will be available to support with any technical issues that arise, so please send them a private message in the chat should you encounter any difficulties during today’s discussion. We will plan on our conversation taking approximately 1 hour, and then completing a brief questionnaire before we finish. Our total time for this focus group will be no more than 90 minutes. Does anyone have any questions before we get started?

**After answering any questions: BEGIN ZOOM RECORDING OR AUDIO RECORDING TO LOCAL COMPUTER**

*Opening/Developing Rapport* [**20 minutes**]

1. Before diving into today’s conversation, I would like for folks to get a better sense of who else is in the room. Could we quickly go around and share your name, which organization you are coming from, and what your role is at the organization?
2. Some clinics are now implementing a status-neutral approach, such that anyone testing HIV-positive is offered ART and anyone testing HIV-negative and at-risk of HIV is offered PrEP. As we discussed earlier, each of your clinics is not currently implementing a status-neutral approach, but intends to do so in the future. What are some reasons that your clinic has not yet implemented a status-neutral approach?
   a. *Follow-up*: How does status-neutral implementation compare with other innovation priorities at your clinics?
3. There is another approach to status-neutral that involves rapid ART initiation and same-day PrEP initiation, called *rapid* status-neutral. For example, this could mean initiating ART within one or seven days or initiating PrEP on the same day as the HIV test. In other words, a *rapid* status-neutral approach would involve integrating both rapid ART and same-day PrEP. What are your initial impressions of this approach to status-neutral?
   a. *Follow-up*: How feasible would a rapid status-neutral approach be for your clinic?

*Section 1: Implementation Barriers & Facilitators* [**30 minutes**]

We will now turn a discussion of barriers and facilitators to introducing a status-neutral approach in your clinics. In other words, we will focus on those things that get in the way of introducing a status-neutral approach or would make the process of introducing it easier. We have developed an illustration that visualizes the sequence of status-neutral implementation at the clinic level. We would like to hear your reflections on it [**SHARE SCREEN**]. This figure was developed from a review of standard operating procedures and consultations with clinicians at organization implementing status-neutral approaches. As a quick orientation, the diagram illustrates the flow of clients through a status-neutral approach, as well as their interactions with different clinic personnel. The process begins with an HIV test, then—depending on the outcome of that test—routes clients to HIV treatment or prevention services. The arrows depict the flow, or sequence, of activities in the status-neutral paradigm. The icons depict the different personnel or roles that may implement the different services illustrated in the diagram. We will give you all a minute or two to digest this image. If the image appears too small on your screen, you can increase the magnification on the bottom right of the shared screen panel within Zoom.

4. What do you see here that aligns well with what you are doing now at your clinics or would be easy to add?
   a. *Follow-up*: What would need to happen to add [*elements mentioned*]?
5. What do you see that would be very different from what you are doing now or would present a challenge to change in your clinics?
   a. *Follow-up*: What would need to happen to change [*elements mentioned*]?
6. What about this *workflow* seems challenging or infeasible to implement in your clinics?
7. What kind of impact or effect do you think implementing a status-neutral approach would have on patient outcomes at your clinic?

*Wrapping Up* [**10 minutes**]

8. We have reached the end of our questions, and I want to thank each of you for your patience, energy, and responses throughout this process. Before we end, I want to ask one final question: what is the most important thing your clinics would need in order to adopt a status-neutral approach?
9. Is there anything I haven’t asked or that hasn’t been discussed today that you feel is important for us to consider?

[**END RECORDING**].
